# n3 and n6 polyunsaturated fatty acids differentially modulate prostaglandin E secretion but not markers of lipogenesis in adipocytes

**DOI:** 10.1186/1743-7075-6-5

**Published:** 2009-01-21

**Authors:** Patrick Wortman, Yuko Miyazaki, Nishan S Kalupahana, Suyeon Kim, Melissa Hansen-Petrik, Arnold M Saxton, Kate J Claycombe, Brynn H Voy, Jay Whelan, Naima Moustaid-Moussa

**Affiliations:** 1 University of Tennessee (UT), Department of Animal Science, Knoxville, TN, USA; 2 University of Tennessee (UT), UT Obesity Research Center, Knoxville, TN, USA; 3 University of Tennessee (UT), Department of Nutrition, Knoxville, TN, USA; 4 Oak Ridge National laboratory, Oak Ridge TN, USA; 5 Michigan State University, Department of Food Science and Human Nutrition, Lansing, MI, USA

## Abstract

A dramatic rise in the incidence of obesity in the U.S. has accelerated the search for interventions that may impact this epidemic. One recently recognized target for such intervention is adipose tissue, which secretes a variety of bioactive substances including prostaglandins. Prostaglandin E_2 _(PGE_2_) has been shown to decrease lipolysis in adipocytes, but limited studies have explored alternative mechanisms by which PGE_2 _might impact obesity, such as adipogenesis or lipogenesis. Studies conducted on *Apc*^Min/+ ^mice indicated that selective inhibition of the cyclooxygenase (COX)-2 enzyme led to significant reductions in fatty acid synthase (FAS) activity in adipose tissue suggesting lipogenic effects of PGE_2_. To further investigate whether these lipid mediators directly regulate lipogenesis, we used 3T3-L1 adipocytes to determine the impact of eicosapentaenoic acid (EPA) and celecoxib on PGE_2 _formation and FAS used as a lipogenic marker. Both arachidonic acid (AA) and EPA dose-dependently increased PGE secretion from adipocytes. AA was expectedly more potent and exhibiting at 150 uM dose a 5-fold increase in PGE_2 _secretion over EPA. Despite higher secretion of PGE by EPA and AA compared to control, neither PUFA significantly altered FAS activity. By contrast both AA and EPA significantly decreased FAS mRNA levels. Addition of celecoxib, a selective COX-2 inhibitor, significantly decreased PGE_2 _secretion (p < 0.05) versus control, and also significantly decreased FAS activity (p < 0.05). Unexpectedly, the combination of exogenous PGE_2 _and celecoxib further decreased the FAS activity compared to PGE_2 _alone or untreated controls. In conclusion, EPA-mediated inhibition of AA metabolism did not significantly alter FAS activity while both AA and EPA significantly decreased FAS mRNA expression. COX-2 inhibition significantly decreased PGE_2 _production resulting in a decrease in FAS activity and expression that was not reversed with the addition of exogenous PGE_2_, suggesting an additional mechanism that is independent of COX-2.

## Background

Several bioactive molecules and hormones have been reported to be secreted by adipose tissue [1,2]. Related to our current work, the secretion of prostaglandins such as prostaglandin E_2 _(PGE_2_) has been shown in human and rodent adipocytes [3-7]. Further, the presence of prostaglandin E receptors (EP_1–4_) in these tissues as well [8-10] has led to research into a possible autocrine or paracrine role for this Arachidonic Acid (AA) metabolite. Several studies reported antilipolytic roles for PGE_2 _in cultured adipocytes and adipose tissue explants [11-13], most likely acting through a G-inhibitory protein-coupled EP_3 _receptor [14], confirming a paracrine/autocrine role of PGE_2 _in adipocytes. Additional roles for PGE_2 _in adipocytes include increased secretion of leptin release from mouse adipose tissue [15] and decreased hepatic lipogenic gene expression [16]. These studies did not, however, address whether PGE_2 _also exerts lipogenic or adipogenic effects in adipocytes.

Several studies have explored regulation of hepatic lipogenesis by polyunsaturated fatty acids (PUFA) [17,18]. However, these effects may be tissue specific as hepatic and adipose lipogenesis may be differentially regulated [19]. Arachidonic acid (AA, 20:4 n-6) has been shown to stimulate glucose intake in adipocytes by increasing glucose receptor levels in the cell membranes [20] potentially increasing substrate availability for de novo lipogenesis. AA is also the preferential substrate for the cyclooxygenase (COX) enzymes, resulting in production of PG and other metabolites [21,22]. In mature adipocytes, PGE_2 _is the primary PG produced from the COX pathway [23-26].

In most cases, AA and dietary eicosapentaenoic acid (EPA, 20:5 n-3) exert opposing effects; however, hepatic lipogenesis is downregulated by both fatty acids [16,27,28]. EPA impacts adipocyte biological functions via two distinct mechanisms. The first is via transcriptional activation of lipogenic and adipogenic genes by binding to nuclear receptors such as PPARgamma. The second mechanism is via direct competition with AA for incorporation into membrane phospholipids and subsequent conversion to eicosanoids including PGs. While the first mechanism has been extensively studied, few studies have addressed the second mechanism in adipocytes. Synthesized or preformed AA is incorporated into the sn-2 position of cell membrane phospholipids and subsequently liberated by phospholipases (cPLA_2_). COX metabolizes AA into PGE_2_, which is then secreted by the cell to act in an autocrine/paracrine fashion via specific EP receptors. EPA competes with AA for incorporation into cell membrane phospholipids and for the active site on the COX enzymes when cPLA_2 _is activated. This competition and the resulting production of PGE_3 _when COX metabolizes EPA, could result in decreased levels of PGE_2_.

Decreased PGs production, and particularly PGE_2_, by COX-2 inhibitors has been demonstrated in adipocytes [4,29-31]. Further, initial in vivo studies demonstrated that inhibition of PGE_2 _synthesis decreased FAS activity in mouse adipose tissue and this was largely reversed by co-treatment with PGE_2 _receptor agonist. We hypothesized that these effects reflected direct actions of PGE_2 _(or its modulators, PUFA or COX ligands) on adipose tissue. Specifically we tested whether manipulation of PGE_2 _levels by addition of EPA (dietary intervention) or selective COX-2 inhibition (pharmaceutical intervention) decreases PGE_2 _production from adipocytes and subsequently downregulates FAS enzyme activity. If AA and EPA modulate FAS activity or expression via changes in PGE_2 _levels, it is then expected that these fatty acids exert opposite effects on FAS expression. We performed a series of experiments in cultured adipocytes which provide compelling evidence that PUFA effects on adipose tissue FAS were independent of changes in PGE levels.

## Materials and methods

### Experiment Design

#### Animal Experiments

Male C57BL/6J *Apc*^Min/+ ^mice (Jackson Labs, Bar Harbor, ME) were obtained at 4–6 weeks of age. Mice were housed in a temperature-controlled room with 14 h periods of light and 10 h periods of darkness and given free access to food and water. The health of the animals was checked daily. Food was withheld overnight prior to sacrifice. All animal procedures were approved by the University of Tennessee Animal Care and Use Committee. Mice were fed a purified AIN-93G powder diet (Dyets, Inc., Bethlehem, PA). Diets containing drug treatments were prepared daily by thoroughly mixing the drugs with the AIN-93G diet to achieve desired drug dosage. Diets were stored at -20°C and all animals were provided fresh food daily. Food consumption was monitored daily and body weights were recorded weekly.

We used the C57BL/6J *Apc*^Min/+ ^mice which we have previously shown to be highly responsive to pharmacological and dietary manipulations of PGE_2 _[32,33]. The experimental design we used has been previously described [32]. Briefly, male C57BL/6J *Apc*^Min/+ ^mice were maintained on the AIN-93G diet until approximately 80 days of age at which time they were randomly assigned to treatment groups consisting of control, E-prostaglandin receptor agonists (EPR-A) [16,16-dimethyl-PGE_2 _and 17-phenyl-trinor PGE_2 _(Cayman Chemical, Ann Arbor, MI), 10 μg each], COX inhibitor (piroxicam [Sigma, St. Louis, MO], 0.5 mg/mouse/day), or piroxicam + EPR-A. The mice were sacrificed at 11–12 weeks of age and epididymal adipose tissue was harvested, weighed, snap frozen in liquid nitrogen and stored at -80°C until further analysis.

#### Cell Culture Experiments

3T3-L1 preadipose cells were purchased from American Type Culture Collection (ATCC, Rockville, MD) and were grown in 100 mm dishes. Culture medium was composed of Dulbecco's modified Eagle's medium (DMEM) supplemented with 10% fetal bovine serum (FBS) and 1% penicillin/streptomycin (P/S). Cells were plated on day 1 (~200,000 cells/100 mm dish) and grown for 3–4 days to confluence. At confluence, media was changed and supplemented with 250 nM Dex, 0.5 mM Mix, and 10 nM insulin for 48 h to induce adipocyte differentiation, after which cells were cultured with regular media supplemented with insulin. Differentiation was considered complete at 5–7 days post-confluence. Twenty-four hours prior to treatment, regular media was replaced with starvation media consisting of DMEM, P/S, and 1% fatty acid-free bovine serum albumin (BSA). Treatment media consisted of starvation media + 10 nM insulin and individual (FA or inhibitor) treatments. Because fatty acids are primarily found in the circulation as bound to albumin and numerous studies have used this form of the fatty acid for cell culture studies [34,35], all fatty acids in our experiments were conjugated with BSA prior to the treatment. Fatty acids were diluted in DMSO, added to the treatment media and incubated in a shaking water bath for 2 hours at 37°C to facilitate this binding. All treatments lasted 48 hours. Fatty acids were purchased from Nu-Check Prep, Inc., Elysian, MN. All studies were conducted in cells that were at least 80–95% differentiated as assessed by lipid accumulation to insure findings are attributed to differentiated rather than undifferentiated fat cells. Detailed experimentation is described below:

#### Dose response studies for AA and EPA on PGE_2_

Dose response studies were conducted to determine physiological EPA dose that would significantly reduce PGE_2 _levels when compared to an equivalent concentration of AA. 3T3-L1 adipocytes were treated with AA or EPA (Nu-Check Prep, Inc., Elysian, MN) in 25, 50, 100, 200, and 500 μM doses. Stock solutions of fatty acids were prepared in dimethyl sulfoxide (DMSO). Treatment time was 48 h for all doses. Cell culture media was collected to measure secreted PGE_2 _levels and cells were harvested to prepare cytosolic extracts used to measure FAS activity.

#### Effects of COX inhibition and various fatty acids and PGE_2 _on FAS

To determine whether treatment of adipocytes with EPA reduces PGE_2 _production by reducing COX enzyme activity, cells were treated with EPA (50 μM and 150 μM), COX-2 inhibitor (celecoxib or CI, 5 μM) (Pharmacia, St. Louis, MO), and a combination of EPA (150 μM) + CI (5 μM). In addition, as a positive control for fat peroxidation, EPA at 150 uM was also added alone to culture media without cells. Treatments lasted 48 hrs, and cell culture media were then collected to measure PGE_2 _levels and cells were harvested for cytosolic extracts. In a second set of experiments, cell cultures were treated with vehicle (DMSO), CI (1 μM), oleic acid (OA, 18:1 n-9), EPA, AA (150 μM each treatment) or AA + EPA (75 μM each fatty acid); media was removed from each dish, and cell cultures were harvested for cytosolic extracts or total RNA.

#### Effect of PGE_2 _supplementation on FAS

To confirm that PGE_2 _was responsible for the decrease in FAS activity seen with selective COX-2 suppression, PGE_2 _was added back to mean levels measured in the control groups of previous experiments. 3T3-L1 cell cultures were treated with vehicle (DMSO), CI (1 μM), PGE_2 _(300 pM) (Cayman Chemical, Ann Arbor, MI), and CI + PGE_2_. 48 hours following treatment, two mL of media was removed from each dish, and cells were harvested for cytosolic extracts or total RNA.

#### FAS assay

The activity of FAS was determined in cytosolic extracts from mouse adipose tissue and cell culture by measuring the rate of oxidation of NADPH as previously described [36]. Briefly, mouse adipose tissue collected from experiment 1 was homogenized on ice in 500 μL of sucrose buffer (pH 7.4). Cell culture plates harvested for cytosolic extracts were washed twice in Hank's balanced salt solution, and then scraped using 350 μL of sucrose buffer. Cell homogenates were sonicated on ice for 5 seconds. Tissue and cell culture homogenates were centrifuged for 1 h (12,000 × g) at 4°C. The supernatant was then removed for analysis of FAS activity and protein concentration.

#### Protein assay

Protein concentration was determined in cytosolic extracts by the method of Bradford [37], using Coomassie blue reagent (Bio-Rad, Melville, NY). Each sample was measured in duplicate using 10 μL of sample and 200 μL of dye in a 96-well plate. After addition of the dye, the samples were incubated for 5 minutes and read in a spectrophotometer at 590 nm. A standard curve was plotted using serial dilutions of a BSA standard of known concentration and sample concentrations were extrapolated based on this standard curve.

#### PGE_2 _assay

Cell culture PGE_2 _concentrations were determined using culture media samples obtained immediately prior to cell harvest. PGE_2 _levels were measured by enzyme immunoassay (EIA) using the Correlate-EIA PGE_2 _Kit (Assay Designs, Ann Arbor, MI) according to the manufacturer's instructions. A standard curve was plotted using serial dilutions of a known concentration of PGE_2 _and sample concentrations were extrapolated based on this standard curve. PGE_2 _EIA assay cross- reactivity for PGE_3 _is reported by the manufacturer to be 16.3%.

#### Real time RT-PCR

Real time RT-PCR was used to determine FAS mRNA expression. Cells harvested for total RNA were scraped in 350 μL of Qiazol lysis reagent (Qiagen, Valencia, CA) and sonicated on ice for 5 seconds. RNA was extracted using the RNeasy™ lipid tissue midi kit (Qiagen) following the manufacturer's protocol. RNA was stored at -80°C for use in real time RT-PCR. Two micrograms of RNA was used to synthesize cDNA. The FAS primers were ordered from Invitrogen (Carlsbad, CA). The sequence of the forward primer was 5'CCCAGAGGCTTGTGCTGACT 3' and the sequence of the reverse primer was 5'CGAATGTGCTTGGCTTGGT 3'. The probe was ordered from Biosearch Technologies, Inc. (Novato, CA). The sequence of the probe was 5'(TET)CCGATCTGGAATCCGCACCGG(TAMRA) 3'. TET is the quencher dye that inhibits the fluorescence of the reporter dye when in close proximity. TAMRA is the reporter dye and is measured at a wavelength of 580 nm. The reactions were performed using the SmartCycler SC1000-1 (one cycle each at 48°C * 1,800 sec and 95°C * 600 sec followed by 40 cycles at 95°C * 15 sec and 60°C * 60 sec) and analysed using SmartCycler software (Cepheid, Sunnyvale, CA).

### Statistical analysis

Statistical analysis for all assays was conducted using SPSS (SPSS for windows, version 12.0, SPSS Inc., Chicago, IL). Data were analyzed for homogeneity of variance and for significant F-ratios between treatment groups using one-way analysis of variance (ANOVA). Post-hoc analysis was conducted using the Bonferroni (equal variance) or Dunnett's T3 (unequal variance) test when significant differences were detected. All values are expressed as mean + SEM. Values of p < 0.05 were considered statistically significant except where otherwise noted.

## Results

### Effect of COX inhibition and EP receptor agonists on FAS activity in mouse adipose tissue

**Figure 1:**
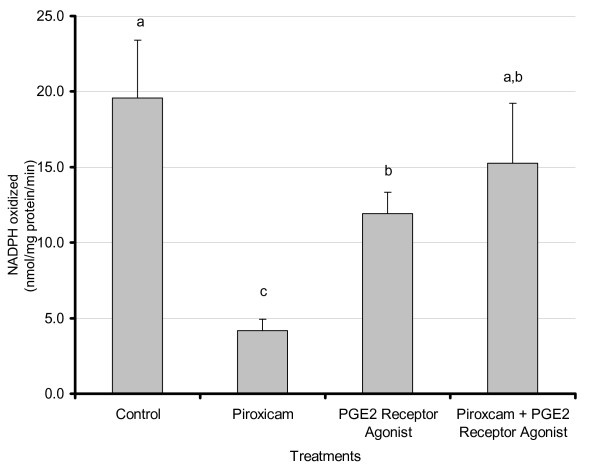
**Effects of piroxicam and PGE_2 _receptor agonists onadipose FAS activity in *Apc*^Min/+ ^mice**. Male C57BL/6J *Apc*^Min/+ ^mice were maintained on the AIN-93G diet until approximately 80 days of age at which time they were randomly assigned to treatment groups. Treatments consisted of control, piroxicam (0.5 mg/mouse/day), EPR-A (16,16-dimethyl-PGE_2 _and 17-phenyl-trinor PGE_2 _-10 μg each), or piroxicam + EPR-A. Mice were sacrificed after 6 days of treatment and epididymal adipose tissue was harvested and snap frozen in liquid nitrogen. Tissue was homogenized in sucrose buffer and cytosolic extracts were analyzed for FAS activity using an activity assay as described in Materials and Methods. For treatments C and P + EPR-A n = 6; for P and EPR-A n = 5. Results represent the mean ± SEM. Values labeled with different letters are significantly different (p < 0.05). Values with the same letters do not differ significantly.

Several studies including ours have previously shown that inhibition of the cyclooxygenase enzymes decrease prostaglandin levels and tumor load in the *Apc*^*Min/+*^ mouse model and that these effects can be reversed by addition of prostaglandin E2 [32]. To determine whether this inhibition also affects fat synthesis, and specifically whether a lipogenic enzyme, FAS, is regulated by prostaglandin levels, adipose tissue from mice treated with vehicle, COX enzymes inhibitor piroxicam and/or EP receptor agonists were studied. As shown in Fig 1, piroxicam significantly decreased FAS activity (4-fold reduction, p < 0.01); similar effects were obtained with another COX inhibitor, sulindac (Data not shown). Combined piroxicam + EP receptor agonists treatments resulted in significant partial restoration of FAS activity (p < 0.02). Treatment with EP receptor agonists alone also resulted in a significant reduction in FAS activity when compared to control (p < 0.02).

### Dose-Response effect of EPA and AA on PGE_2_ secretion and FAS activity in 3T3-L1 adipocytes

**Figure 2:**
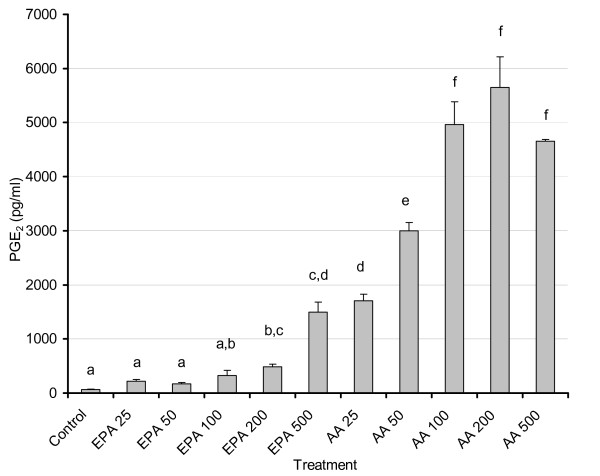
**Dose-response effects of AA and EPA on PGE_2 _levels in 3T3-L1 adipocytes**. 3T3 L-1 adipocytes were grown 6–7 days post-confluence, and starved for 24 h in serum-free media containing FA-free BSA. Fatty acids were incubated in media containing FA-free BSA for two hours at 37°C in a shaking water bath prior to treatment. Treatment consisted of AA and EPA at 25, 50, 100, 200, and 500 μM concentrations. After 48 hours, media was removed and stored at -80°C until PGE_2 _levels were measured as described in the Materials and Methods. For treatments AA/EPA 500 n = 5; control (C) and AA/EPA 25 n = 10; AA/EPA 50, 100, 200 n = 15. Values labeled with different letters are significantly different (p < 0.05). Results represent the mean ± SEM. Values with the same letters do not differ significantly.

Consistent with studies that have shown the displacement of AA by EPA in tissue phospholipids [33,38-40], the levels of PGE_2 _were significantly lower when EPA was added to the cell culture media compared to equivalent concentrations of AA (Fig. 2). As expected, addition of AA led to a powerful dose-dependent increase in PGE_2_ production. The two highest doses of EPA (200 μM and 500 μM) resulted in PGE_2_ levels that were significantly higher than control (p < 0.03), although this is most likely attributed to the formation of PGE_3_[39,41]. However, at the doses tested above, FAS enzyme activity did not exhibit any significant changes from control, following AA or EPA treatments except for AA at 500 μM (p < 0.03) which was significantly higher than control (data not shown).

### Effect of EPA versus COX inhibition (CI) on PGE levels in 3T3-L1 adipocytes

**Figure 3:**
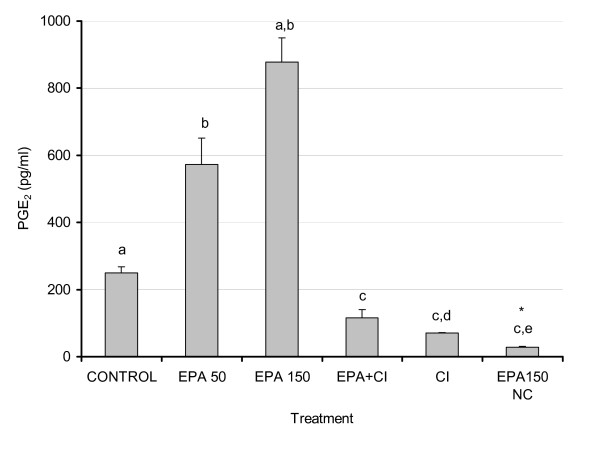
**Effects of EPA and COX-2 inhibition on secreted PGE_2 _levels from 3T3-L1 adipocytes**. 3T3 L-1 adipocytes were grown 6–7 days post-confluence, and starved for 24 h in serum-free media containing FA-free BSA. Fatty acids were incubated in media containing FA-free BSA for two hours at 37°C in a shaking water bath prior to treatment. Treatment consisted of EPA (50 and 150 μM), EPA (150 μM) + CI (5 μM), CI (5 μM) and EPA (150 μM) in media without cells. After 48 hours, 2 mL of media was removed and stored at -80°C. Media PGE_2 _levels were analyzed using EIA as described in the Materials and Methods. Results represent the mean ± SEM with a number of treatments n = 3–4. Values labeled with different letters are significantly different (p < 0.05). Values with the same letters do not differ significantly. * Denotes culture media exposed to cell culture conditions without cells.

Antagonistic effects of EPA and AA have been extensively studied and reported and in most cases, EPA effects mimic effects of COX inhibition. Increasing doses of EPA as reported in Fig. 2 led to increased PGE_2_ levels, presumably due to the formation of PGE_3_ (41) as the antibody had a weak cross reactivity of PGE_3_ with the PGE_2_. Addition of CI + EPA (150 μM) led to a significant reduction in PGE_2_ (p < 0.01) (Fig. 3), indicating that the PGE formation is for the most part an enzymatic process. Addition of EPA in the absence of cultured cells led to extremely low levels of PGE confirming that the observed effects were not a consequence of non-specific lipid peroxidation and that increased PGE_2_ levels measured in EPA treated cell cultures, supporting the likelihood of PGE_3_ formation, which is detectable with the PGE_2_ immunoassay used. Our data consistently show that the PGE_2_ formation is an enzymatic process, and since no exogenous AA was added to the cells these data collectively support the idea that production of PGE_3_ is responsible for the increases seen in measured PGE_2_ production versus control.

Effect of selective COX-2 inhibition and different fatty acids on PGE_2_ levels, FAS activity and FAS mRNA levels in 3T3-L1 adipocytes

**Figure 4:**
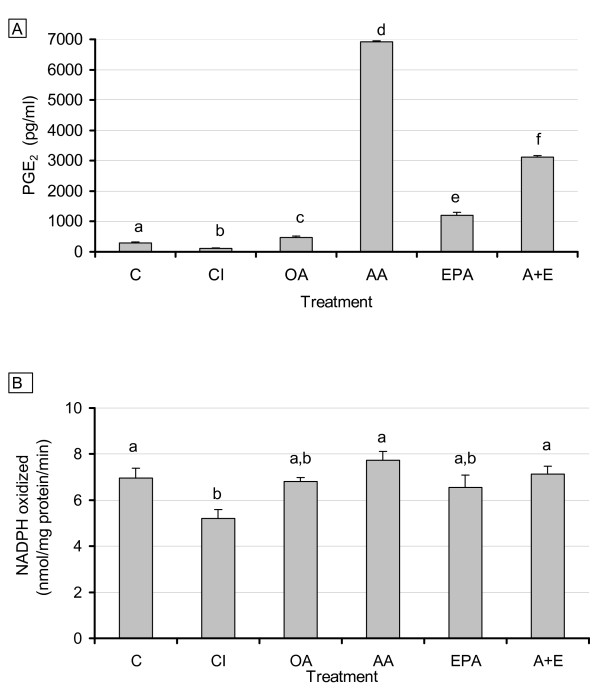
**Effects of COX-2 inhibition and EPA addition on PGE_2 _levels (4A) and FAS activity (4B)**. 3T3 L-1 adipocytes were grown 6–7 days post-confluence, and starved for 24 h in serum-free media containing FA-free BSA. Fatty acids were incubated in media containing FA-free BSA for two hours at 37°C in a shaking water bath prior to treatment. Treatment consisted of CI (1 μM), OA, EPA, AA (150 μM each treatment), and AA + EPA (75 μM each FA). After 48 hours, 2 mL of media was removed, and cells were scraped using 350 μL of sucrose buffer, sonicated, and centrifuged for 1 hour. Media and cytosolic extracts were stored at -80°C. Media PGE_2 _levels and FAS activity were measured as described in the Materials and Methods. For all treatments n = 10. Results represent the mean ± SEM. Values labeled with different letters are significantly different (p < 0.05). Values with the same letters do not differ significantly.

As shown above, AA significantly increased PGE_2 _levels in 3T3-adipocytes and to a lesser extent, EPA also increased PGE levels. PGE_2 _levels were effectively reduced compared to control by the addition of CI (p < 0.001). All fatty acid treatments resulted in measured increases in concentrations of PGE compared to control, with responsiveness increasing in the order of OA (p < 0.05), < EPA < AA + EPA < AA (all p < 0.001) (Fig. 4A). Consistent with the decrease in PGE_2 _levels by CI, FAS enzyme activity was significantly decreased by COX inhibition (p < 0.05); however, EPA and OA exhibited only trends in decreasing FAS activity without reaching statistical significance (Fig. 4B).

**Figure 5:**
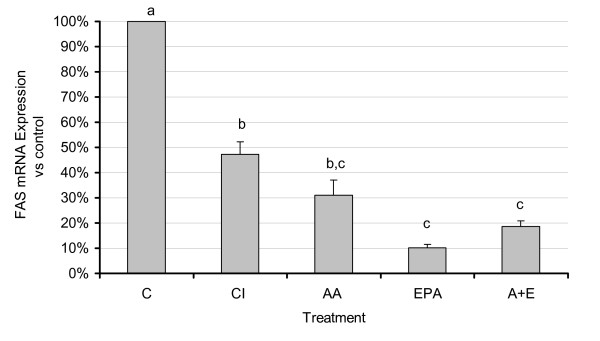
**Effects of n3 and n6 PUFA and COX-2 inhibition on FAS mRNA expression**. 3T3 L-1 adipocytes were grown 6–7 days post-confluence, and starved for 24 h in serum-free media containing FA-free BSA. Fatty acids were incubated in media containing FA-free BSA for two hours at 37°C in a shaking water bath prior to treatment. Treatment consisted of CI (1 μM), EPA and AA (150 μM each treatment), and AA + EPA (75 μM each FA). After 48 hours, cells were scraped using 350 μL of Qiazol lysis reagent, and total RNA was extracted using the RNeasy™ lipid tissue midi kit (Qiagen) following the manufacturer's protocol. RNA was stored at -80° for further analysis. Real time RT-PCR analysis was performed as described in the materials and methods. For all treatments n = 6. Results represent the mean ± SEM. All values were significantly different than control, and values labeled with different letters are significantly different (p < 0.05) from each other. Values with the same letters do not differ significantly.

Expression of FAS mRNA showed significant changes with COX inhibition and with AA and EPA treatment compared to control (p < 0.05) (Fig. 5). The level of reduction of FAS mRNA by the FA treatments correlated well with the degree of unsaturation and chain length and may therefore reflect a non-specific PUFA effect as previously reported for PUFA effects on hepatic lipogenic genes [42,43].

### Effect of exogenously added PGE_2 _on selective COX-2 inhibition of endogenous PGE_2 _secretion, FAS activity, and FAS mRNA levels in 3T3-L1 adipocytes

**Figure 6:**
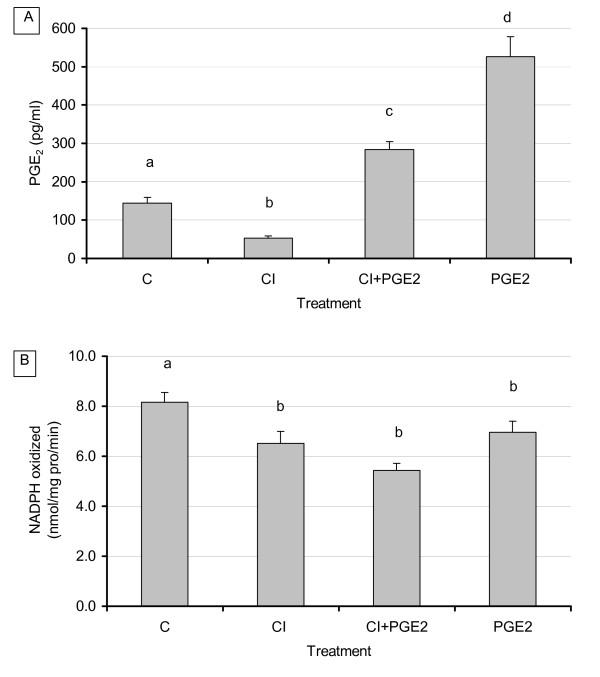
**Effect of exogenous PGE_2 _and COX inhibition on adipocyte PGE_2 _secretion (6A) and FAS activity (6B)**. 3T3 L-1 adipocytes were grown 6–7 days post-confluence, and starved for 24 h in serum free media containing FA free BSA. Treatment consisted of CI (1 μM), PGE_2 _(300 pM), and CI + PGE_2_. After 48 hours, 2 mL of media was removed and cells were scraped using 350 μL of sucrose buffer, sonicated, and centrifuged for 1 hour. Media and cytosolic extract were stored at -80° Media PGE_2 _levels and FAS activity were measured as described in the Materials and Methods. For all treatments n = 8. Results represent the mean ± SEM. Values labeled with different letters are significantly different (p < 0.05). Values with the same letters do not differ significantly.

As demonstrated in our previous experiments, addition of CI resulted in a significant decrease in PGE_2 _production (p < 0.04). Addition of PGE_2 _to cells treated with CI restored PGE_2 _levels and resulted in significantly higher levels versus CI treatment alone or versus control (p < 0.02) (Fig. 6A). Addition of PGE_2 _to cultures without CI resulted in the expected significant increases of PGE_2 _over control levels (p < 0.02). The level of PGE_2 _added was based on the mean of previously measured levels of PGE_2 _secreted by 3T3-L1 control groups (300 pM). Analysis of FAS enzyme activity and mRNA expression, however, produced unexpected results regarding CI and PGE_2 _interactions. Treatment with CI resulted in a consistent decrease in FAS enzyme activity while combined CI and exogenous PGE_2 _resulted in a significant and further decrease in FAS activity (p < 0.03) rather than the hypothesized reversal of CI inhibition by PGE_2 _(Fig. 6B). Similar effects were also observed on FAS mRNA (not shown). Addition of PGE_2 _alone, in the absence of CI, exerted no significant changes on FAS.

## Discussion

We and others have previously shown that adipocytes secrete significant amounts of prostaglandins [3-7,30]. Further, it is well established that PGE_2 _decreases lipolysis in adipocytes [11-14]. Our current study addressed whether PGE_2 _also induces lipogenic activities in adipocytes, which could further enhance their hypertrophic effects in these cells. We have previously reported responsiveness of the *Apc*^Min/+ ^mice to changes in prostaglandin levels [35]. In this study we tested whether changes in prostaglandins modulated fatty acid synthesis in adipose tissue of these mice. We demonstrated that inhibition of the COX enzymes by piroxicam (or other COX inhibitors, data not shown) decrease FAS activity (Fig. 1); this effect was reversed by admnistering mice EP receptor agonists. These results demonstrate that a lipogenic and receptor-mediated effect of PGE_2_, which coupled with its previously reported antilipolytic effects [11-14] likely favor triglyceride storage. To further test direct effects of PGE_2 _manipulation in adipocytes, we used 3T3-L1 adipocytes and used dietary polyunsaturated fatty acids (AA and EPA) as well as pharmacological means (COX inhibition) to modulate prostaglandin levels. Our goal was to test whether changes in PGE_2 _levels led to parallel changes in FAS activity or expression.

We report here dose-dependent increases in PGE_2 _levels with AA and EPA treatments (p < 0.001) and, as expected, AA exhibited a more potent induction of PGE_2 _secretion versus EPA or control. Very limited information exists in the literature regarding physiological levels of EPA, but they are unlikely to approach those of AA due to large difference in their levels in membrane phospholipids. Studies conducted on postmenopausal women fed fish oil found the EPA levels of plasma lipids was 750 μM [36,37]. Our dose response studies indicate that despite the clearly powerful effect of AA in increasing PGE_2 _(compared to EPA), the latter was also able to significantly elevate PGE_2 _levels especially at the 200 and 500 uM doses. Doses at 500 uM and above visibly impacted cell viability and morphology (data not shown). Based on the manufacturer's information for cross-reactivity (and confirmed in our laboratory), the assay also detects PGE_3_ (cross reactivity with PGE_2 _antibody), which may explain at least in part increased PGE_2 _levels with EPA. Although the binding affinities of AA and EPA for both isoforms of COX are equivalent (*Km *= 5 μM), COX-1 and -2 oxygenate EPA at ~10% and ~35%, respectively, compared to the rate for AA when added exogenously to cultured cells. Compared to AA, EPA is a poorer substrate for COX-1 *in vivo*[44]. Complicating this issue further is the fact that very little is known concerning the actions of PGE_3 _and its subsequent down stream signaling [42]. Studies in NIH 3T3 fibroblasts found that PGE_3 _activated the same signaling pathways as PGE_2_, but with much less efficiency [3]. This would imply that high levels of PGE_3_ might duplicate the actions of PGE_2_, suggesting there would be a point of diminishing returns with EPA supplementation and thus the importance of using appropriate low doses for experimentation and supplementation. It is also possible that PGE_3_ may bind to EP receptors with affinities that differ significantly from PGE_2 _[42], whereby the type and number of receptors in adipocytes would also influence the impact of EPA supplementation. While it is important to point out these differences in PGE_2 _vs. PGE_3 _formation, the main focus of this paper is primarily on the role of PG and COX in modulating fatty acid synthesis.

Based on our dose response data, we chose to use intermediate dosage of 150 μM for the rest of our experiments. This dose is also consistent with the findings of other studies [3,35], which demonstrated the ability to manipulate secreted PGE_2 _by using EPA to compete with AA for incorporation into membrane phospholipids and subsequent PG production.

PGE_2 _levels in the CI treatment group were significantly lower than controls. Although the celecoxib preferentially inhibits COX-2, no studies have shown whether it reduces PGE_2 _levels in adipocytes. Most published evidence indicates that inducible enzyme COX-2 is not expressed at physiologically relevant levels in mature adipocytes under normal conditions [31,45]. However, since we used a CI dose (1 μM) similar to that of IC_50 _(1.2 μM [46]), it is plausible that constitutively expressed COX-1 activity was also inhibited at this dose. Using FAS as a marker of adipocyte lipogenesis, our data showed decreases in FAS activity with the CI treatment. In agreement with our finding, another study also showed that a non-specific COX inhibitor, aspirin, also decreases lipogenesis or levels of triacylglycerols in adipocytes [31].

The overall comparison of PGE levels for the AA, EPA, and AA + EPA treatment groups correlated well with previously published data measuring tissue concentrations of these fatty acids in mice fed diets supplemented with these fatty acids, although the end points measured and experimental models were different in our study compared to those reported [33,40,47].

Treatment of adipocytes with either EPA or AA elicited significant reductions in FAS mRNA compared to control, consistent with previously reported inhibition of FAS message by PUFA (36) in a degree of unsaturation and chain length-dependent manner [17,48]. Therefore, regulation of FAS expression in adipocytes by PUFA is independent of changes in prostaglandin levels. Alternatively, PG could be directly controlling FAS gene expression via a receptor-mediated mechanism. Such opposing effects may be explained by direct transcriptional effects of EPA and AA on the FAS gene. Indeed, this theory is in line with results from Deng et al. showing that degree of unsaturation correlated highly with suppression of the SREBP-1c promoter [39], the main transcription factor regulating FAS and other lipogenic genes. However, it is worth noting that since mRNA stability was not measured in these experiments, it is also possible that increased stability was responsible for the discrepancy between decreased FAS mRNA expression and small changes in FAS activity in response to PUFA treatments. It is also possible that this discrepancy is due to a longer half-life of the FAS protein such that we were unable to detect changes in enzyme activity within our treatment times (24–48 hours). Indeed, previous studies have shown that changes in FAS mRNA half-life depend on cell culture treatments and state of differentiation [49,50]. Another possibility is that PGE_2 _treatments stimulate leptin secretion (as previously documented Fain et al [15]) and elevated leptin levels may subsequently decrease lipogenesis [51]. Thus, decreased FAS expression may indirectly reflect effects of PGE_2 _mediated by leptin [52,53]. Unfortunately, due to the very low levels of leptin secretion in 3T3-L1, we were not able to assess regulation of leptin in these cells. Additional possible mechanisms may involve the antithetic actions of the EP receptors. Long et al. investigated the role of COX mediated products of AA metabolism on regulation of glucose transporter 4 (GLUT 4). They found that a 50-fold increase in endogenous PGE_2 _or exposure to 10 μM exogenous PGE_2 _resulted in an increase in cAMP concentrations, consistent with activation of the EP_2_/EP_4 _receptor [54]. Additionally, studies using the specific COX-2 inhibitor NS-398 on cortical collecting duct cells found that NS-398 treatment increased EP_3 _and EP_4 _receptor expression 3-fold [55]. Although the concentration of exogenous PGE_2 _added in our treatments was much lower, preliminary gene expression studies demonstrate that celecoxib also influenced the expression of EP receptors (specifically EP_4_, data not shown), which may differ from effects of aspirin or other non-specific COX inhibitors. An increase in EP_4 _receptors and the resulting increase in cAMP would activate a pathway that would oppose the decrease in cAMP responsible for the PGE_2 _mediated decrease in lipolysis. Further work with EP receptor concentration and mechanism of action is necessary to delineate the exact role of each receptor in regulating adipocyte metabolism.

One surprising finding is that PGE_2 _recapitulated the decrease of FAS activity and expression by COX inhibition but that combined PGE_2 _and celecoxib treatments further decreased FAS. These results may be due to and complicated by changes in EP receptor expression and signaling with PGE_2 _addition. It is also possible that the pathways mediating PGE_2 _effects via its receptors are different from those affected by COX inhibition. Further, COX inhibition was more potent than EPA in reducing FAS, possibly due to higher levels of the two PGE isoforms in the presence of EPA compared to control.

Overall, our studies demonstrated the ability to pharmacologically decrease production of PGE_2 _using the selective COX-2 inhibitor celecoxib, which resulted in a significant reduction in lipogenic enzyme activity. The use of EPA was also shown to result in lower production of PGE_2 _when compared to AA treatment. FAS mRNA expression was decreased by FA treatment in a manner similar to the PUFA effects seen in the liver [16,27,28]. Given that SREBP1c, an insulin responsive transcription factor, mediates PUFA regulation of hepatic lipogenic genes, it is possible that PUFA regulation of adipocyte metabolism modulates insulin sensitivity. Indeed, in the absence of insulin, our cells expressed significantly higher PGE_2 _levels than in the presence of insulin (data not shown).

Low PGE_2 _levels led to decreased lipogenesis and thus a reduction in PGE_2 _levels would result in less inhibition of lipolysis, which coupled with reported antilipogenic effects of EPA and other polyunsaturated fatty acids would still favorably impact adipose tissue levels and result in decreased adiposity.

Further experimentation is required to determine the mechanism of action of celecoxib and EPA versus PGE_2 _in adipose tissue. Additional data on the receptor affinities and actions of PGE_3_, although difficult to approach at this time given the lack of data in this area, are necessary in order to understand the mechanism mediating EPA and PGE_3 _effects on lipid metabolism.

This study also brings to light the need for further studies on the function and impact of PGE_3_ in adipocytes to determine if it does in fact elicit the same responses as PGE_2_. If this is the case, then it may indicate a point of diminishing benefit and the need to specify a consumption range instead of recommending minimum consumption levels. Understanding the mechanisms involved with EPA metabolism takes on further importance with the FDA move to allow qualified health claims for omega-3 fatty acids [56], as this will most certainly bring more attention to these fatty acids and increase their consumption even further.

## Competing interests

The authors declare that they have no competing interests.

## Authors' contributions

PW carried out PG and FAS analysis studies in 3T3-L1 adipocytes and first draft of the manuscript; YM performed analyses of mouse adipose tissue; NK contributed to data analysis and manuscript writing; SK contributed to cell culture studies; MHP carried out the mouse studies; AS carried out and reviewed statistical analysis of the data; KC contributed to experimental design in cultured cells and to manuscript writing; BHV contributed to cell culture design and manuscript writing; JW carried out experimental design of the mouse studies and contributed to design of the cell culture experiment and manuscript review and editing; NMM conceived and coordinated this study, carried out the experimental design of the cell culture experiments, trained PW, YM and NK and finalized the manuscript for submission; All authors read and approved the final manuscript version.
